# Prevalence and Risk Factor Analysis of Feline Blood-Borne Pathogens in Bangkok and Vicinities, Thailand

**DOI:** 10.1155/vmi/6882793

**Published:** 2025-09-18

**Authors:** Nonsee Rodmanee, Duangchanok Umnuayyonvaree, Morakot Kaewthamasorn, Vachira Hunprasit, Sukullaya Ritthikulprasert

**Affiliations:** ^1^Department of Veterinary Medicine, Faculty of Veterinary Science, Chulalongkorn University, Bangkok 10330, Thailand; ^2^Center of Excellence in Veterinary Parasitology, Faculty of Veterinary Science, Chulalongkorn University, Bangkok 10330, Thailand

**Keywords:** Anaplasmataceae, *Bartonella*, blood-borne infection, hemotropic mycoplasmas, piroplasm

## Abstract

Feline blood-borne pathogens are important infectious agents of cats that can cause subclinical to severe disease. Awareness of the risks associated with transfusing contaminated blood can reduce morbidity in recipients. Understanding the prevalence and risk factors of these infections is crucial for identifying pathogens that should be screened in feline blood donors. A total of 410 blood samples from client-owned cats were collected between 2018 and 2021 across veterinary hospitals in the Bangkok Metropolitan Area. Conventional PCR was used to detect hemotropic mycoplasmas, *Bartonella* spp., Anaplasmataceae, and piroplasms. Overall, 20.5% of samples were tested positive for at least one pathogen. Hemotropic mycoplasmas were the most detected agents (16.3%), with “*Candidatus* Mycoplasma haemominutum” being predominant, followed by *Mycoplasma haemofelis*. DNA of *Bartonella* spp. was identified in 5.4% of samples, specifically *Bartonella henselae* and *Bartonella clarridgeiae*. No samples tested positive for Anaplasmataceae or piroplasms. Univariable and multivariable logistic regression analyses revealed that male sex, Domestic Shorthair breed, anemia, clinical illness, and increasing age were significant risk factors for hemotropic mycoplasma infection, but kittens were less likely to be infected. Additionally, infection with feline immunodeficiency virus was associated with a higher likelihood of hemotropic mycoplasma positivity in samples collected during 2020-2021. No significant risk factors were identified for *Bartonella* spp. infection. In conclusion, the findings underscore the necessity of screening feline blood donors for hemotropic mycoplasma and *Bartonella* spp. Clinically healthy, female, purebred cats without outdoor access cats are recommended as preferred blood donor candidates, given their lower risk of hemotropic mycoplasma infection.

## 1. Introduction

Hemotropic mycoplasmas (hemoplasmas), *Bartonella* spp., and tick-borne pathogens including *Ehrlichia* spp., *Anaplasma* spp., *Babesia* spp., and *Cytauxzoon* spp. are recognized as causative agents of feline blood-borne infections. Among these, hemotropic mycoplasmas are considered the most clinically significant. Domestic cats serve as natural reservoirs for *Bartonella* spp., which are also potentially zoonotic. Although *Cytauxzoon* spp. can cause fatal disease in felines, its incidence appears to be geographically restricted. The remaining pathogens are generally less pathogenic, with most naturally infected cats exhibiting subclinical infections.

Due to the potential for transmission through blood transfusions, routine screening for hemotropic mycoplasmas and *Bartonella* spp. is mandatory for all feline blood donors. Screening for other tick-borne pathogens is recommended in endemic regions [1]. Feline blood-borne infections have been reported globally, with prevalence rates varying across different geographical areas. In Thailand, infections with hemotropic mycoplasmas and *Bartonella* spp. are commonly observed among client-owned cat populations [2–6]. In contrast, infections with *Babesia vogeli* are sporadic, and *Anaplasma platys* was documented in only one feline case [7, 8].

However, data regarding the prevalence of other blood-borne infections remain limited. Therefore, the present study aims to provide updated epidemiological information and identify potential risk factors associated with these infections in client-owned cats.

## 2. Materials and Methods

### 2.1. Animals

This study was approved by the Institutional Animal Care and Use Committee of the Faculty of Veterinary Science, Chulalongkorn University (Approval No. 2031069). The biosafety protocol was reviewed and approved by the Institutional Biosafety Committee of the same faculty (Approval No. 2031045). Blood samples were collected from 289 client-owned cats, irrespective of age, sex, or breed, between 2020 and 2021. These cats were recruited from the Small Animal Teaching Hospital at Chulalongkorn University, as well as from private veterinary clinics and hospitals located in Bangkok and its vicinities. Cats that had received doxycycline or fluoroquinolones within 30 days prior to presentation or had undergone a blood transfusion with any blood product were excluded from the study. Written informed consent was obtained from all owners prior to participation. A detailed clinical history and physical examination were conducted for all cats. Attending veterinarians completed standardized questionnaires, which gathered information on lifestyle (e.g., outdoor access, household size, and contact with other animals), medical history (e.g., reason for visit and clinical signs on the day of presentation), physical examination findings (including body temperature), previous transfusion history, and preventive healthcare practices (e.g., vaccinations and ectoparasite control). Results from blood tests requested by the attending veterinarians were added to the data collection form. In addition, 112 archived feline blood samples collected between 2018 and 2019 from a previous study on feline blood typing were included for analysis [9]. Only limited data on signalment and medical history were available for these archived cases. Information on blood type and packed cell volume was accessible for all archived samples.

### 2.2. Sample Collection and Blood Tests

Blood samples were collected via venipuncture from peripheral or cephalic veins and transferred into ethylene diamine tetra-acetic acid (EDTA) and heparinized tubes at the time of presentation. Heparinized samples were used for biochemical analyses as requested by the attending veterinarians. EDTA-anticoagulated blood was divided into two aliquots for complete blood count and polymerase chain reaction (PCR) assays. Retroviral testing was performed using commercially available test kits for feline leukemia viral antigen and feline immunodeficiency viral antibody. A minimum of 200 μL of blood was reserved for PCR testing. These samples were immediately frozen at −20°C until further analysis.

### 2.3. Molecular Analysis

Conventional PCR assays (Table 1) were performed based on established protocols described in previous studies [10–12]. Primers targeting piroplasms in this study were previously designed based on sequences from piroplasms infecting buffaloes and were validated for amplification of *Babesia* deoxyribonucleic acid (DNA) in canine blood samples [13]. The PCR reactions were run in a PCR thermocycler (SureCycler 8800, Agilent Technologies). PCR products were visualized by agarose gel electrophoresis using gels with a concentration of 2%–3%. PCR-positive samples underwent DNA extraction and sequencing. Nucleic acids were extracted using a commercial extraction kit (DNeasy® Blood & Tissue Kit, Qiagen, Hilden, Germany), following the manufacturer's protocol. DNA sequences were aligned manually using BioEdit Sequence Alignment Editor Version 7.2.5 and compared with reference sequences from the NCBI GenBank™ database using the Basic Local Alignment Search Tool (BLAST).

### 2.4. Statistical Analysis

Statistical analyses were conducted using SPSS for Windows (version 22; SPSS Inc., Chicago, IL, USA). Epidemiological variables obtained from physical examinations, questionnaires, and hematological parameters were reported for each pathogen and analyzed to identify potential risk factors. Initially, all variables were assessed for multicollinearity. Categorical variables were evaluated using Pearson's chi-square test, followed by Phi coefficient and Cramér's V test to assess the strength of association. Continuous variables were analyzed using Pearson's correlation test. In cases where multicollinearity was identified between two variables, the variable with greater biological plausibility was retained for further analysis. Univariable logistic regression was subsequently performed to assess the association between each variable and pathogen detected. Variables with a *p* value less than 0.2 were included in the multivariable logistic regression model. To enhance clinical applicability, continuous variables such as age, body temperature, and hematological parameters were categorized prior to inclusion in the model. Variables with a *p* value less than 0.05 in the multivariable analysis were considered statistically significant risk factors for infection. Interactions between potential confounding variables were not assessed in this analysis.

## 3. Results

The prevalence distribution of each infection is presented in Table 2. Positive PCR results were observed in 84 of 410 (20.5%) blood samples. Among these, 67 cats (16.3%) tested positive for feline hemoplasmas. Fifty-eight cats (14.1%) were infected with “*Candidatus* Mycoplasma haemominutum” (“*C.* M. haemominutum”), while 9 cats (2.2%) were infected with *Mycoplasma haemofelis* (*M. haemofelis*). Of 410 cats, 22 cats (5.4%) were tested positive for *Bartonella* spp. infection. Of these, *Bartonella henselae* (*B. henselae*) was identified in 16 cats (3.9%), and *Bartonella clarridgeiae* (*B. clarridgeiae*) were detected in 6 cats (1.5%).

No PCR-positive results were detected for *Anaplasma* spp., *Ehrlichia* spp., *Babesia* spp., or *Cytauxzoon* spp. No co-infections involving multiple species within the same genus were found. However, co-infections involving both hemoplasmas and *Bartonella* spp. were identified. Specifically, two cats were co-infected with “*C.* M. haemominutum” and *B. henselae*, while three additional cats were co-infected with “*C.* M. haemominutum” and *B. clarridgeiae.*

Among the hemotropic mycoplasma samples, PCR products of approximately 190 bp in size showed high sequence similarity to “*C.* M. haemominutum,” with sequence identities ranging from 97.95% to 100% when compared to reference sequences in GenBank (accession numbers: MF281072, MN240865, and MN240867). Two 190-bp samples yielded weaker sequencing signals, resulting in lower confidence scores (e.g., higher e-values). However, their sequences still suggested probable identification as “*C.* M. haemominutum,” with 94.57%–96.67% identity to sequences MN240864 and MN240865. The PCR products approximately 170 bp in size were identified as *M. haemofelis*, showing 99.41%–100% sequence identity with reference sequences AM748929 and MN240855.

For the *Bartonella* PCR-positive samples, those producing PCR bands of approximately 172 bp were identified as *B. henselae*, with sequence identities ranging from 99.4% to 100% when compared to the reference sequence CP072904 in GenBank. The remaining *Bartonella*-positive samples, which produced a PCR product of approximately 157 bp, were identified as *B. clarridgeiae*, showing 100% identity to the reference sequence CP116497. No other *Bartonella* species—such as *Bartonella koehlerae* or *Bartonella vinsonii* subsp. *berkhoffii*—were detected in the sequenced samples.

Univariable logistic regression analysis identified several significant risk factors associated with infection by any hemoplasma species (Table 3). Cats with hemoplasma infection were significantly more likely to be of the Domestic Shorthair (DSH) breed (odds ratio [OR] = 4.279; 95% confidence interval [CI]: 1.786–10.252), clinically ill (OR = 2.649; 95% CI: 1.449–4.842), male (OR = 2.822; 95% CI: 1.500–5.308), anemia (OR = 2.306; 95% CI: 1.201–4.427), or older (OR = 1.135; 95% CI: 1.037–1.243). In contrast, kittens were significantly less likely to be infected (OR = 0.172; 95% CI: 0.041–0.726). In the multivariable logistic regression analysis (Table 4), three factors remained significantly associated with hemoplasma infection: being a DSH (OR = 4.344; 95% CI: 1.784–10.574), male sex (OR = 2.856; 95% CI: 1.486–5.489), and kitten status, which continued to show a protective effect (OR = 0.203; 95% CI: 0.047–0.874).

For “*C.* M. haemominutum” infection, the risk factors identified by univariable logistic regression analysis were similar to those associated with any hemoplasma infection (Table 5). Significant risk factors included being a DSH (OR = 4.410; 95% CI: 1.708–11.389), senior age category (OR = 3.333; 95% CI: 1.092–10.171), anemia (OR = 2.553; 95% CI: 1.277–5.103), male sex (OR = 2.534; 95% CI: 1.313–4.888), clinical illness at presentation (OR = 2.461; 95% CI: 1.300–4.659), and increasing age (OR = 1.161; 95% CI: 1.057–1.276). In contrast, kitten status was found to be a protective factor (OR = 0.098; 95% CI: 0.012–0.726). Multivariable logistic regression analysis (Table 6) revealed that male sex (OR = 2.597; 95% CI: 1.275–5.291), DSH breed (OR = 3.721; 95% CI: 1.396–9.915), and clinical illness (OR = 2.217; 95% CI: 1.105–4.445) remained significantly associated with infection. Kitten status continued to demonstrate a protective effect (OR = 0.109; 95% CI: 0.014–0.820).

In addition to the absence of certain tests or data from samples collected during 2018–2019, a significant finding emerged from the 2020–2021 cohort. Feline immunodeficiency virus (FIV) infection was identified as a significant risk factor for both any hemoplasma infection and “*C.* M. haemominutum” infection. In the univariable logistic regression analysis, the odds ratios for FIV infection were 11.887 (95% CI: 3.548–39.831) for any hemoplasma infection and 10.692 (95% CI: 3.378–33.847) for “*C.* M. haemominutum” infection. The multivariable logistic regression analysis confirmed these findings, with odds ratios of 8.824 (95% CI: 2.336–33.324) for any hemoplasma infection and 11.476 (95% CI: 3.002–43.874) for “*C.* M. haemominutum” infection.

None of the factors were identified as significant risk factors in the analysis of any blood sample group or for the species of *Bartonella*.

## 4. Discussion

In our study, the prevalence of feline hemotropic mycoplasmas was found to be 16.3%. In Thailand, previous studies have reported prevalence rates of 22.9% and 27.7% in client-owned cats [2, 5]. Semidomesticated cats, such as those in monasteries or temples, have been shown to have higher prevalence rates of 36.9% and 38.06% [3, 14]. Furthermore, one study reported a prevalence of 9.6% for feline hemoplasmosis in apparently healthy cats [15]. While the prevalence observed in our study was lower than that in previous reports for client-owned cats, it is noteworthy that more recent blood samples showed a higher positivity rate for hemoplasma detection compared to earlier samples. The prevalence of hemoplasmosis may vary depending on factors such as the study population, the timing of sample collection, and the presence of various risk factors among the sampled cats.

Likewise, “*C.* M. haemominutum” was the most prevalent species, followed by *M. haemofelis*. This finding aligns with the trends observed in previous prevalence studies of feline hemoplasmosis in Thailand. We did not detect “*C.* M. turicensis” in any of the hemoplasma-positive samples. Although “*C.* M. turicensis” has been identified in cats in Thailand, it has the lowest prevalence, reported at 0.9% [3, 5]. All samples submitted for DNA sequencing that exhibited a 190-bp PCR product were identified as “*C.* M. haemominutum.” This band size is expected to differentiate “*C.* M. haemominutum” from other hemoplasma species [11]. Additionally, all DNA sequences corresponding to the 170-bp band size were identified as *M. haemofelis*.

We found that male cats were significantly more likely to be infected with hemotropic mycoplasmas. The odds ratios of 2.856 for any hemoplasma species infection and 2.597 for “*C.* M. haemominutum” infection are consistent with those reported in previous studies, where odds ratios typically range from approximately 2 to 4 for any hemoplasma species infection and from 1.94 to 4.84 for “*C.* M. haemominutum” infection [14, 16–33]. Male cats have been prone to initiate fights with other cats, and they are also more frequently diagnosed with aggression as a behavioral problem compared to female cats [34, 35]. Direct transmission of hemoplasmosis through biting is highly possible, with risk factors including the presence of abscesses, being bitten, or sustaining bite wounds from fights, which reflect the aggressive behavior commonly exhibited by male cats [31, 36].

DSH cats were also identified as a significant risk factor in our study, with odds ratios of 4.344 for any hemoplasma species infection and 3.721 for “*C.* M. haemominutum” infection. Previous studies have reported odds ratios ranging from 3.03 to 5.55 for hemoplasma infections in DSH cats [22, 29, 37, 38]. One study found significant associations between DSH cats and infection with any hemoplasma species or “*C.* M. haemominutum,” whereas another reported significance only for “*C.* M. haemominutum” infection [25, 29]. It is suggested that DSH cats are more likely to have outdoor access compared to purebred cats and may be adopted from environments where they previously lived in close contact with infected animals [38]. Furthermore, purebred cats are often raised in controlled environments, such as breeding catteries, where higher standards of care may reduce exposure to the pathogens, potentially explaining their lower risk of infection.

Being a kitten was identified as a significant protective factor against feline hemoplasmosis, including both any hemoplasma species and “*C.* M. haemominutum” infections. Several studies have similarly reported that kittens are at significantly lower risk of hemoplasma infection [3, 14, 26, 39]. Age was associated with hemotropic mycoplasma infection in the univariable analysis; however, this association did not remain significant in the multivariable analysis. Additionally, univariable logistic analysis identified senior age as a specific risk factor for “*C*. M. haemominutum” infection.

Previous studies have identified mature adult and senior cats as being at increased risk of hemoplasma infection [3, 14, 26, 31, 32, 37, 39]. The observed association between advancing age and susceptibility to hemoplasma infection is likely multifactorial. Older cats may serve as chronic carriers—particularly in cases involving “*C.* M. haemominutum” infections—and are more likely to have experienced cumulative exposure to infection sources over time [40]. Additionally, they may be affected by immunosuppressive conditions, including chronic diseases, neoplasia, or retroviral infections, which can compromise immune function, thereby increasing susceptibility to infection and reducing the capacity to eliminate the pathogen. This, in turn, may contribute to the higher detection rates of hemoplasma in older feline populations.

In this study, cats exhibiting clinical signs of illness were found to be at greater risk of infection with “*C.* M. haemominutum.” Compared to apparently healthy cats, those presenting with clinical signs had a higher likelihood of being infected with “*C.* M. haemominutum,” consistent with findings from previous studies [29, 41–43]. However, other studies have reported no significant association between clinical signs and “*C.* M. haemominutum” infection [40, 44]. This discrepancy may suggest that “*C.* M. haemominutum” is either capable of inducing clinical disease in some cases or is present as a comorbidity alongside other illnesses. For infections caused by any hemoplasma species, the presence of clinical signs was a significant risk factor in univariable analysis, but this association did not remain significant in multivariable analysis. Most previous studies have also failed to find a clear association between clinical illness and infection with hemoplasmas in general [27, 31, 32, 45, 46]. Conversely, a few studies have reported that symptomatic cats are more likely to be infected with hemotropic mycoplasmas [29, 42]. Differences in these findings may be attributed to variations in hemoplasma species distribution and pathogenicity, as some studies may have included a high proportion of infections caused by less virulent species, thereby masking potential associations with clinical disease.

Cats that tested positive for FIV antibodies using a commercial test kit were significantly more likely to be infected with “*C.* M. haemominutum” or any hemoplasma species. The odds ratios for “*C.* M. haemominutum” and any hemoplasma infection were 11.476 and 8.824, respectively. For hemoplasma infections in general, previous studies have reported odds ratios ranging from 2.4 to 26.8 in FIV-seropositive cats [16, 18, 22, 23, 26, 27, 29, 31, 32, 42, 47–49]. The most plausible explanation for this co-infection is the shared route of transmission between FIV and hemoplasmas, primarily through aggressive interactions such as biting or fighting, as well as through transfusion of contaminated blood products [50]. Furthermore, cats infected with FIV may be immunocompromised due to viral-induced immunosuppression, which can impair their ability to clear hemoplasma infections, thereby increasing the likelihood of persistent infection.

Not only has FIV infection been identified as a risk factor for hemotropic mycoplasma infection, but it is also considered a comorbidity. Both infections may remain subclinical in cats, allowing asymptomatic carriers of hemoplasmas and FIV to transmit these pathogens to recipients, potentially leading to clinical consequences [1]. In a previous study, all co-infected cats exhibited no signs of systemic illness; however, hemotropic mycoplasma infection in cats with FIV-induced immunosuppression may present with more severe clinical manifestations, including anemia, delayed recovery, and an increased risk of recurrent or chronic illness [16]. Further studies are required to identify prognostic factors associated with FIV infection in cats co-infected with hemotropic mycoplasmas.

For *Bartonella* spp., the prevalence of infection among cats was 5.4%. In Thailand, reported prevalence rates based on conventional PCR detection in client-owned cats range from 0% to 17% [2, 4, 15, 51, 52]. Additionally, *Bartonella* spp. prevalence has been reported at 2.83% in stray cat populations and 9.4% in mixed populations [6, 52]. The prevalence observed in our study falls within the range of previously reported values for feline populations in Thailand. Our findings also indicate that *B. henselae* is the most common species detected, with the prevalence of 5.4%, which aligns with previous studies reporting prevalence rates between 5.7% and 11.1% [2, 6]. The less common species is *B. clarridgeiae*, with reported prevalence ranging from 0.7% to 5.9% [2, 4, 6, 53]. In contrast, *Bartonella koehlerae* and *Bartonella vinsonii* subsp. *berkhoffii* were not amplified in our study. These latter two species are considered rare in cats but have been previously reported in Thailand [2, 6, 54].

None of the evaluated factors in this study were found to be significantly associated with *Bartonella* spp. infection. Age, outdoor access, ownership status (client-owned or stray), flea infestation, co-infection, and ectoparasitic prevention were factors included in the present analysis that have been reported to be significantly associated with *Bartonella* infection [2, 6, 38, 44, 45]. The inability to identify these risk factors in this study may be attributed to several reasons. First, the number of PCR-positive samples for *Bartonella* spp. was relatively low, potentially limiting the statistical power needed to detect significant associations. Second, some variables were observed only in either the PCR-positive or PCR-negative groups, preventing the calculation of odds ratios. For example, *Bartonella*-positive cats were exclusively found in the group without flea infestation, and none of the *Bartonella*-positive cats exhibited hyperthermia. Lastly, the number of cats exhibiting potential risk factors was limited—for instance, only 42 cats were reported to receive regular ectoparasite prevention, and only 10 cats had confirmed flea infestations. Therefore, further investigations are warranted to identify potential risk factors for *Bartonella* infection.

This study represents the first investigation into the prevalence of feline anaplasmosis and ehrlichiosis in Thailand. However, *Anaplasma* spp. and *Ehrlichia* spp. were not detected in any of the samples. To date, there is only one published report of feline anaplasmosis in Thailand, involving a cat infected with *A. platys* [7]. Additionally, informal interviews with attending veterinarians and veterinary technicians revealed that feline anaplasmosis has been suspected in clinical settings, with some laboratory findings reporting the presence of *A. platys*-like inclusion bodies in platelets on blood smears (unpublished data). Despite reports of *A. platys* infection in cats from other parts of the world, the infection may be rare in Thai feline population. It is possible that the bacteremia level of *A. platys* in infected cats is low and falls below the detection threshold of the conventional PCR assay used in this study. Unfortunately, no feline cases with confirmed *A. platys* infection were available for assay validation; as such, positive control material was derived from canine samples. A zero-prevalence finding for feline anaplasmosis and ehrlichiosis remains plausible, as several studies conducted in other countries have also failed to detect these pathogens in cats. However, further research is needed to assess the diagnostic sensitivity of conventional PCR for the detection of *Anaplasma* spp. and *Ehrlichia* spp. in feline samples. In suspected clinical cases, the use of nested PCR or real-time PCR may be recommended due to their enhanced sensitivity compared to conventional assays.

In Thailand, the prevalence of feline babesiosis has been reported in two studies involving stray cats; however, to date, no such studies have been conducted in client-owned cats [8, 14]. Additionally, feline cytauxzoonosis has never been identified through molecular detection in Thailand. This study is the first to investigate the prevalence of *Babesia* spp. in client-owned cats and to assess the potential for detecting *Cytauxzoon* spp. using a PCR assay targeting *Babesia* DNA. None of the cats in our study tested positive for *Babesia* or *Cytauxzoon* spp. by PCR. The absence of positive results may be explained by the very low prevalence of these pathogens in the study population. Our findings are consistent with several international studies that also failed to detect feline babesiosis [41, 55–58]. Although sporadic cases of feline babesiosis have been reported, more recent studies in countries such as Italy and Spain have similarly failed to detect any infections [23, 44, 49, 59–64]. In contrast, studies from Brazil have shown variable results, with some reporting the presence of feline babesiosis while others did not detect any cases [58, 65–70]. As with conventional PCR assays for Anaplasmataceae, the sensitivity of the assay used in this study may not be sufficient to detect low levels of parasitemia. Although a canine blood sample was used as a positive control, no *Babesia* DNA was amplified from any feline samples, indicating the need for further evaluation and optimization of the assay. Enhancing the diagnostic sensitivity may improve detection rates, especially in cases with low pathogen loads. Furthermore, we recommend the use of nested PCR or quantitative PCR in future studies to distinguish between true pathogen absence and potential under-detection resulting from methodological limitations.

Based on the findings of this study, it is recommended that feline blood donors should be screened for hemotropic mycoplasma and *Bartonella* spp. infections prior to blood collection. In Thailand, the prevalence of feline hemotropic mycoplasmas and *Bartonella* infections ranges approximately from 10.7% to 38% and 0% to 17%, respectively, depending on the sampled population. In contrast, other pathogens investigated in this study, including *Anaplasma* spp., *Ehrlichia* spp., *Babesia* spp., and *Cytauxzoon* spp., may not require routine screening prior to blood donation due to their very low prevalence or apparent absence in the region.

Feline blood donors are required to be apparently healthy, and any cats exhibiting signs of illness should be excluded from donation. However, in this study, hemoplasma and *Bartonella* DNA were detected in the blood of some cats that appeared clinically healthy. The prevalence of hemoplasma and *Bartonella* infections among these cats was 10.8% and 7.0%, respectively. Furthermore, ideal feline blood donors should be young adults, typically between 1 and 7 years of age. Univariable logistic analysis revealed that older cats were at a significantly higher risk of hemoplasma infection. Among cats aged 1–7 years, the infection rates were 16.7% for hemoplasma species and 6.2% for *Bartonella* species. These findings underscore the importance of screening all feline blood donor candidates for hemoplasma and *Bartonella* infections, regardless of their apparent health status.

Following the selection of feline blood donors based on signalment and absence of clinical signs, candidates must undergo hematological testing. Any cat presenting with abnormalities in the blood profile—particularly anemia—should be excluded from donation. However, even in the absence of anemia, cats may still serve as asymptomatic carriers of hemoplasma and *Bartonella* spp. In this study, hemoplasma and *Bartonella* DNA were detected in nonanemic cats, with prevalence rates of 11.7% and 7.4%, respectively. Additionally, infections with feline immunodeficiency virus and feline leukemia virus have been identified as risk factors for hemoplasma infection; however, these pathogens can be readily identified using commercially available diagnostic test kits.

Considering all identified risk factors for feline hemotropic mycoplasmas infection in this study, the feline blood donor candidates with the lowest likelihood of hemoplasma infection are those that are female, purebred, and have no history of outdoor access. As previously noted, these candidates should also be clinically healthy, within the young adult age range (1–7 years), and free from laboratory abnormalities. Moreover, additional factors such as blood type and crossmatch compatibility must also be considered when selecting appropriate feline blood donors.

In routine blood bank settings, blood products are collected from feline donors and stored for future use. Under these circumstances, blood-borne pathogens can be screened using PCR testing, and results are typically available prior to transfusion. However, in emergency situations, blood is often collected from feline donors and transfused immediately into recipients, which may preclude the availability of PCR test results at the time of transfusion. Unless feline blood donors undergo regular screening for blood-borne pathogens, it is recommended that blood products be routinely subjected to PCR testing to evaluate the potential risk of transmitting infectious agents to recipients.

This study has several limitations. First, not all cats included were ideal candidates for blood donation. Our exclusion criteria were limited to cats with a history of antimicrobial use or prior blood transfusion. Due to time constraints, some cats enrolled in the study may not have been appropriate blood donors, including those exhibiting clinical signs of illness, as well as kittens and senior cats. Furthermore, feline blood samples from confirmed cases of *Anaplasma* spp., *Ehrlichia* spp., *Babesia* spp., or *Cytauxzoon* spp. infections were not available for the validation of our PCR assay. Additionally, data on the sensitivity of the PCR assay for detecting piroplasm species are lacking. Although the low natural prevalence of these pathogens suggests our results may reflect the true infection rates, in cases of high clinical suspicion, more sensitive methods—such as nested PCR or real-time PCR—may be warranted to enhance detection.

## 5. Conclusions

Hemoplasma and *Bartonella* spp. infections are commonly detected in client-owned cats, whereas other blood-borne pathogens, including *Anaplasma* spp., *Ehrlichia* spp., *Babesia* spp., and *Cytauxzoon* spp., were not identified in any of the cats examined. Major risk factors associated with hemoplasma infection include male sex, DSH breed, presence of clinical illness, and concurrent FIV infection. In contrast, kittens appear to be less likely infected. No significant risk factors for *Bartonella* infection were identified in this study. When selecting feline blood donors, preference should be given to cats that are female, purebred, and strictly indoor, when feasible. This study supports the implementation of routine screening for hemoplasma and *Bartonella* spp. in all feline blood donor candidates prior to transfusion in order to minimize the risk of transmitting these pathogens to recipients.

## Figures and Tables

**Table 1:** Primer sets for PCR amplification and PCR conditions.

Pathogen	Gene	F/R	Nucleotide	PCR condition	Product size (bp)	Reference
Feline hemotropic mycoplasma	*16S* rRNA	F	ACGAAAGTCTGAT GGAGCAATA	95°C 2 min, 45 cycles (95°C 1 min, 60°C 1 min, 72°C 30 s), 72°C 5 min	170, 193	[11]
		R	ACGCCCAATAAAT CCGRATAAT			

*Bartonella*	*16S-23S ITS* rRNA	F	(C/T)CTTCGTTTCTC TTTCTTCA	95°C 2 min, 45 cycles (95°C 1 min, 60°C 1 min, 72°C 30 s), 72°C 5 min	154–260	[12]
		R	AACCAACTGAGCT ACAAGCC			

Piroplasmida	*18S* rRNA	F	GGTACCYACAGA AGAAGTCC	95°C 5 min, 35 cycles (94°C 30 s, 55°C 30 s, 72°C 90 s), 72°C 5 min	345	[10]
		R	TAGCACTCATCGT TTACAGC			

Anaplasmataceae	*16S* rRNA	F	GCAAATTACCCAATCC TGACACAGG	94°C 2 min, 40 cycles (98°C 10 s, 60°C 30 s, 68°C 90 s), 68°C 5 min	1221	[13]
		R	CCGAATAATTCACCGGA TCACTCG			

**Table 2:** Prevalence of infection.

Infection	*N* (prevalence)
Any infection	84 (20.5%)
Any hemoplasma infection	67 (16.3%)
“*C.* M. haemominutum” infection	58 (14.1%)
*M. haemofelis* infection	9 (2.2%)
Any *Bartonella* species infection	22 (5.4%)
*B. henselae* infection	16 (3.9%)
*Bartonella clarridgeiae* infection	6 (1.5%)
*Ehrlichia* or *Anaplasma* infection	0 (0%)
*Babesia* or *Cytauxzoon* infection	0 (0%)
Co-infection between “*C.* M. haemominutum” and *B. henselae*	2 (0.5%)
Co-infection between “*C.* M. haemominutum” and *B. clarridgeiae*	3 (0.7%)

**Table 3:** Univariable analysis of any hemoplasma species infection.

Variables	Category	PCR positive	PCR negative	Odds ratio	95% CI	*p* value
Age	Continuous	—	—	1.135	1.037–1.243	0.006

Generation	Kitten	2	53	0.172	0.041–0.726	0.017
	Young adult	46	229	1.245	0.663–2.340	0.495
	Mature adult	8	30	1.469	0.639–3.379	0.365
	Senior	5	10	2.786	0.918–8.458	0.071

Sex	Male	50	181	2.822	1.500–5.308	0.001
	Female	14	143	Reference		

Breed	DSH	57	222	4.279	1.786–10.252	0.001
	Others	6	100	Reference		

Hematocrit	Continuous	—	—	0.965	0.928–1.003	0.070

Anemia	Anemia	22	72	2.306	1.201–4.427	0.012
	Nonanemia	22	166	Reference		

Sickness	Sick	46	143	2.649	1.449–4.842	0.002
	Apparently healthy	17	140	Reference		

**Table 4:** Multivariable analysis of any hemoplasma species infection.

Variables	Odds ratio	95% CI	*p* value
DSH	4.344	1.784–10.574	0.001
Male	2.856	1.486–5.489	0.002
Kitten	0.203	0.047–0.874	0.032

**Table 5:** Univariable analysis of “*C*. M. haemominutum” infection.

Variables	Category	PCR positive	PCR negative	Odds ratio	95% CI	*p* value
Age	Continuous	—	—	1.161	1.057–1.276	0.002

Generation	Kitten	1	54	0.098	0.013–0.726	0.023
	Young adult	39	236	1.110	0.576–2.138	0.756
	Mature adult	8	30	1.778	0.767–4.120	0.180
	Senior	5	10	3.333	1.092–10.171	0.034

Sex	Male	43	188	2.534	1.313–4.888	0.006
	Female	13	144	Reference		

Breed	DSH	50	229	4.410	1.708–11.389	0.002
	Others	5	101	Reference		

Hematocrit	Continuous	—	—	0.962	0.923–1.002	0.062

Anemia	Anemia	20	74	2.553	1.277–5.103	0.008
	Nonanemia	18	170	Reference		

Sickness	Sick	39	150	2.461	1.300–4.659	0.006
	Apparently healthy	15	142	Reference		

**Table 6:** Multivariable analysis of “*C*. M. haemominutum” infection.

Variables	Odds ratio	95% CI	*p* value
Male	2.597	1.275–5.291	0.009
DSH	3.721	1.396–9.915	0.009
Sick	2.217	1.105–4.445	0.025
Kitten	0.109	0.014–0.820	0.031

## Data Availability

The data that support the findings of this study are available from the corresponding author upon reasonable request.
